# Injectable “Skin Boosters” in Aging Skin Rejuvenation: A Current Overview

**DOI:** 10.1055/a-2366-3436

**Published:** 2024-11-13

**Authors:** Nark-Kyoung Rho, Hyun-Seok Kim, Soo-Young Kim, Won Lee

**Affiliations:** 1 Department of Dermatology Center, Leaders Aesthetic Laser and Cosmetic Surgery Center, Seoul, Republic of Korea; 2 Invited Faculty of Minimal Invasive Plastic Surgery Association, Seoul, Republic of Korea; 3 Department of Plastic Surgery, Kim Hyun Seok Plastic Surgery Clinic, Seoul, Republic of Korea; 4 Department of Plastic Surgery, Ichon Plastic Surgery Clinic, Seoul, Republic of Korea; 5 Scientific Faculty of Minimal Invasive Plastic Surgery Association Seoul, Republic of Korea; 6 Department of Plastic Surgery, Yonsei E1 Plastic Surgery Clinic, Anyang, Republic of Korea

**Keywords:** amino acid, botulinum neurotoxin, collagen, hyaluronic acid, intradermal injection, needle-free jet injector, poly-(lactic acid), polycaprolactone, polydeoxyribonucleotide, skin booster

## Abstract

Aging-related changes in the skin, such as dullness, dehydration, and loss of elasticity, significantly affect its appearance and integrity. Injectable “skin boosters,” comprising various biological materials, have become increasingly prominent in addressing these issues, offering rejuvenation and revitalization. This review offers a comprehensive examination of these injectables, detailing their types, mechanisms of action, and clinical uses. It also evaluates the evidence for their effectiveness and safety in treating age-related skin alterations and other conditions. The goal is to provide an insightful understanding of injectable skin boosters in contemporary dermatological practice, summarizing the current state of knowledge.

## Introduction

**Fig. 1:**
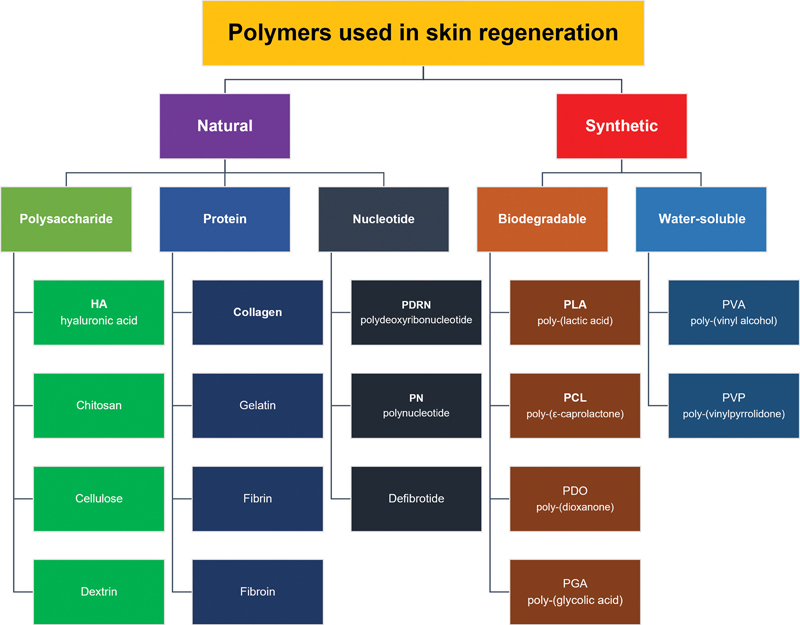
Classification of different polymers utilized as skin-boosting agents, encompassing those in clinical practice and under investigation.

Aging manifests as progressive skin deterioration, weakening its structure and aesthetic appeal with dullness, dehydration, and loss of elasticity. To combat these age-related changes and treatment side effects, “skin boosters” have gained traction in aesthetic procedures, 1 focusing on improving skin quality. These bioactive materials, known for their minimal invasiveness, safety, and short recovery time, vary widely in composition. This review focuses exclusively on injectable skin boosters, leaving out topical types. These boosters are primarily classified by their biocompatible polymer ingredients, whether naturally or synthetically derived. Figure 1 presents a range of polymer-based skin boosters, both currently in clinical use and under research, offering a comprehensive perspective.

## Natural Biopolymers

### Hyaluronic Acid

Attaining hydration is crucial for augmenting the skin's inherent luminosity and overall visual appearance, as it correlates closely with the skin's radiance and can be assessed through visual, tactile, and biomechanical means. Hyaluronic acid (HA) is pivotal in augmenting skin hydration. Its role in dermal hydration has made HA a preferred choice for injectable skin-boosting treatments. Originally termed from a commercial HA product, Restylane Skinboosters (Galderma, Lausanne, Switzerland), HA is the most thoroughly used agent in this category. Predominantly used in small particle-sized cross-linked gels, HA is a glycosaminoglycan abundantly present in the dermal extracellular matrix (ECM), exhibiting remarkable hydrophilic properties, binding water up to 1,000 times its volume, thereby maintaining skin viscoelasticity, hydration, and fiber integrity. Adequate HA levels in the dermis correlate with firm skin, optimal turgor, and minimized fine lines. 2 As a natural, nontoxic product to dermal fibroblasts, 3 HA's water retention capacity is proportional to its concentration, 4 with studies suggesting optimal ranges from 12 to 20 mg/mL for skin quality enhancement. 5 6 7

**Fig. 2:**
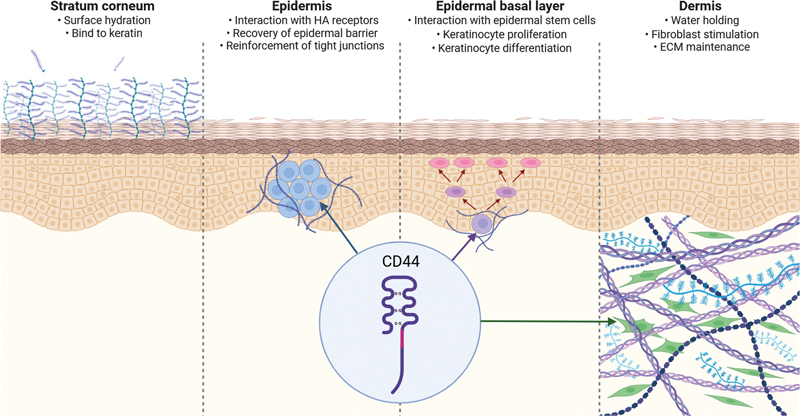
Depiction of the suggested mechanisms of action of hyaluronic acid as a skin-boosting agent, delineating its effects across distinct skin layers. ECM, extracellular matrix; HA, hyaluronic acid.

Research reveals that HA stimulates collagen I synthesis in fibroblasts 8 and enhances the structural support of the ECM via mechanical stretching from HA injections. This process activates the TGF-β signaling pathway, leading to increased type I collagen production. 8 9 10 HA interacts with hyaluronan receptors CD44 and CD168, promoting fibroblast migration and proliferation 9 11 ( Fig. 2 ), and inhibits collagenase activity, reducing collagen breakdown, and enhancing skin smoothness. 8

A 2018 consensus highlights cross-linked HA-based skin boosters as the preferred first-line hydration treatment, 12 effective alone or combined with other agents. 13 Intradermal HA injections target fine wrinkles and delicate areas like crow's feet, with specific techniques applicable for less cross-linked gels, smaller particle sizes, or lower HA concentrations. 14 Cross-linked HA shows diffuse, homogeneous restoration and maintenance of dermal ECM and fibers, differing from HA used for volume replacements. 5 Kim's study 15 demonstrated that intradermal cross-linked HA injections improved skin texture, significantly improved skin roughness, reduced electric resistance, and thickened the face and hand dermis by approximately 4%, unlike subdermal injections that only replaced fluid volume without improving skin texture, suggesting the superficial, intradermal injection technique is effective for dermal rejuvenation. However, non-cross-linked HA has shown inconsistent results, possibly due to rapid degradation by endogenous hyaluronidase without cross-links. 13 This may result in insufficient or unsustainable outcomes, with differences in particle size and HA concentration potentially contributing to the variability observed. 16 Non-cross-linked HA can lead to heightened stratum corneum hydration and a relative decrease in transepidermal water loss (TEWL). 5

Intradermal injection of cross-linked HA can sometimes result in the formation of “beads” or “papules,” a phenomenon influenced by both the product and skin characteristics. This issue is frequently observed on the cheek skin, particularly on the lateral parts. To prevent this issue, it is advisable to utilize lightly cross-linked HA, administer small bolus injections, and avoid excessively superficial placement.

### Polynucleotide

**Fig. 3:**
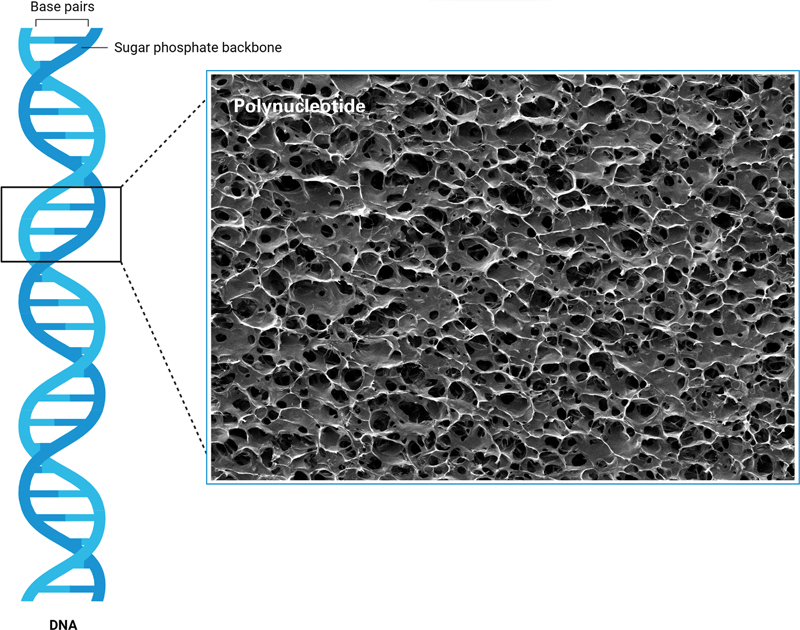
An image captured through a scanning electron microscope displaying a commercial polynucleotide gel product (REJURAN, PharmaResearch, Gyeonggi-do, Korea), highlighting a consistent porosity indicative of a quality tissue scaffold.

Polydeoxyribonucleotide (PDRN) is a complex of deoxyribonucleotide polymers, with chain lengths ranging from 50 to 2,000 base pairs, primarily derived from *Oncorhynchus mykiss* (salmon trout) or *Oncorhynchus keta* (chum salmon) sperm DNA, yielding over 95% pure active substances alongside inactivated peptides and proteins. 17 PDRN acts as a selective adenosine A2A receptor in medicine 18 and facilitates tissue repair, and anti-inflammatory effects, and has been applied in treating degenerative joints and diabetic foot ulcers. 19 Polynucleotide (PN), a related substance, consists of high-molecular-weight DNA chains from salmon or trout gonads, offering superior viscoelasticity and water-binding properties compared with PDRN. 20 PN forms a durable three-dimensional porous structure ( Fig. 3 ), providing ECM support and tissue scaffolding, making it ideal for skin rejuvenation. 19 It also exhibits anti-inflammatory effects in vivo. 18 PLINEST (Mastelli, Sanremo, Italy) marks the initial commercial PN-based injectable medical device in Europe, and REJURAN (PharmaResearch, Gyeonggi-do, Korea) is currently serving as the prominent injectable PN product in Asian regions. Both are designed for direct intradermal injection.

PN is recognized as a safe option for skin rejuvenation, owing to its high immunological safety profile. It functions as a biostimulator, enhancing collagen production, elasticity, and hydration. 21 22 A survey of 235 board-certified Korean dermatologists specializing in cosmetic procedures, revealed that 88% use PN injections in their cosmetic practices. 19 A study involving Korean women who received four intradermal PN injections at 2-week intervals showed marked improvements in pore size, skin thickness, skin tone, melanin levels, wrinkles, and sagging, with no severe side effects reported. 23 European research with 20 patients demonstrated significant dermal quality enhancement and atrophic acne scar reduction from PN injections, confirming its safety and effectiveness as a single treatment. However, this calls for larger, longer-term randomized studies for more conclusive evidence. 24 Furthermore, PN/PDRN offers immunomodulatory and antioxidative benefits. 21 25 26 A survey of 557 Korean aesthetic physicians found widespread use and effectiveness of intradermal PN injections for facial erythema arising from inflammatory dermatosis and repeated laser treatments. 27

Several clinical studies have assessed the effectiveness of injectable PN products for periorbital crow's feet lines. An animal experiment and a clinical trial with 72 Korean patients showed significant improvements in elasticity, collagen composition, skin surface roughness, and wrinkle depth following intradermal PN injections, outperforming non-cross-linked HA. 28 These findings were replicated in another randomized, pair-matched, and active-controlled study using the same products. 29 Additionally, a study using a three-dimensional skin surface scanner on 30 Korean subjects reported improved scores in crow's feet grading, wrinkles, texture, pores, depression, and skin redness after PN injections. 30

Combining PN with HA has been shown to more effectively activate fibroblasts than either substance alone in vitro. 31 Research using acellular porcine dermis and PN-enriched HA showed superior results in accelerating healing and promoting reepithelialization, myofibroblast activation, neoangiogenesis, and collagen deposition compared with polyurethane foam in chronic ulcer treatment. 32 Experts recommend using PN and HA together in the same device for an enhanced hygroscopic effect. 33 In Korea, over 50% of dermatologists using PN as an injectable skin booster frequently combine it with HA. 19

In Korea, the standard clinical practice for skin rejuvenation involves administering 2 mL of PN every 3 to 4 weeks across three to four sessions. Intradermal needle injections are evenly distributed across the face, with a focus on problematic areas. 27 In Europe, a consensus suggests that after three consecutive treatment sessions, spaced 3 weeks apart, the effects typically last between 6 and 12 months. 21 22 Additionally, using an intradermal PN injection as a “priming” step before skin treatments like lasers, fillers, and surgeries has been shown to enhance results. 19 33

### Collagen

Collagen, a prevalent natural polymer, is widely used in tissue engineering, particularly for skin regeneration due to its unique properties. 34 Historically, collagen products have been used in clinical settings as scaffolds for tissue replacement, notably in skin substitutes and dermal fillers, capitalizing on their natural abundance in collagen-rich tissues. However, due to limitations like inferior mechanical properties and susceptibility to enzymatic degradation in skin, the use of injectable collagen as volume fillers has shifted in favor of HA fillers. Despite this, intradermal collagen injections offer unique benefits in skin regeneration, including proliferation, biocompatibility, flexibility, and controlled degradation. 35 Renewed interest in intradermal collagen injections for regenerative dermatology has emerged among dermatologists. The latest innovations include atelocollagen, derived from nonhuman sources, which is a low immunogenic form of collagen obtained by removing N- and C-terminal telopeptides responsible for human antigenicity. 36 This involves treating collagen with type I pepsin to remove the telopeptides, 37 preserving the native protein structure and functionality. 38 LAETIGEN (D-Med Resources, Gyeonggi-do, Korea), a new porcine atelocollagen product, exemplifies these advancements, designed specifically for intradermal application to enhance aging skin quality.

Despite the potential of injectable collagen scaffolds in enhancing skin quality, there remains a need for further clinical and laboratory research. Critical questions, such as whether collagen's therapeutic effects are due to fibrosis induced by injections or the inherent properties of the collagen itself, are yet to be fully addressed. The role of collagen fragmentation in producing peptide cytokines, known as “matrikines,” offers an interesting avenue for investigation, as these can significantly influence the remodeling of the ECM. 39 Delving deeper into the complex interactions among cells, mechanical forces, and collagen in aging skin is crucial for driving future advancements in this field.

### Platelet-rich Plasma and Stromal Vascular Fraction

Platelet-rich plasma (PRP) derived from peripheral blood and stromal vascular fraction (SVF) from adipose tissue are renowned autologous skin-boosting agents in regenerative medicine and surgery, recognized for their remarkable tissue regeneration capabilities. PRP combined with SVF has shown promise in treating intractable dermatoses 40 and facilitating breast reconstruction. 41 Intradermal injections of PRP and SVF have demonstrated efficacy in treating acne scars with acceptable safety profiles. 42 Recent trials have shown that injecting SVF along with PRP into the scalps of patients with androgenetic alopecia can significantly increase hair density within 6 to 12 weeks, although further research is needed to determine the optimal treatment regimen. 43 However, the present review primarily focuses on skin-boosting “products,” and a detailed review of PRP and SVF in regenerative dermatology and surgery is beyond its scope. For more information on PRP and SVF, readers are referred to studies mentioned as references. 44 45

## Biodegradable Synthetic Polymers

Beyond natural materials like collagen and HA, several biocompatible synthetic polymers have been explored for their capacity to stimulate fibroblasts and promote neocollagenesis. 46 Biodegradable polymers, including polylactic acid (PLA), poly-(ε-caprolactone; PCL), and polydioxanone (PDO), demonstrate superior longevity and enhanced collagen synthesis in vivo, compared with HA. 47 These properties make them increasingly popular in dermatology and plastic surgery as injectable options. 48 49 Understanding the distinct interactions of these polymers with biological systems is key to optimizing their practical applications, as they are often engineered to improve physiological conditions and biological functions. 50 Table 1 summarizes the characteristics and profiles of these biodegradable polymers currently approved for injection-based applications.

**Table 1:** Biodegradable synthetic polymer products approved for injection applications

Main ingredient	Form	Storage	Reconstitution before use	Products
PLLA	Lyophilized powders	Vial	Yes	SCULPTRA (Galderma, Switzerland) OLIDIA (PRP Science, Korea) GANA FILL (GANA R&D, Korea)
PDLLA	Lyophilized powders	Vial	Yes	AESTHEFILL (Regen Biotech, Korea) JUVELOOK (VAIM, Korea)
PCL	Gel-form suspension	Prefilled syringe	No	ELLANSE (AQTIS Medical, The Netherlands) LAFULLEN (Samyang Holdings, Korea) GOURI (DEXLEVO, Korea)
PDO	Lyophilized powders	Vial	Yes	ULTRACOL (Ultra V, Korea)

Abbreviations: PCL, polycaprolactone; PDLLA, poly-(D,L)-lactic acid; PDO, polydioxanone; PLLA, poly-(L)-lactic acid.

### Polylactides

PLA, a thermoplastic aliphatic polyester, varies in properties based on its stereochemical forms. 51 Used as a nonsurgical rejuvenation method, PLA is injected subcutaneously to gradually create volume over time offering an alternative to facial fat grafting. 52 In medical and surgical applications, two primary types of PLA are used: poly-(L)-lactic acid (PLLA) and poly-(D,L)-lactic acid (PDLLA), a copolymer of the L- and D-forms of PLA. The stereochemistry of PLA isomers significantly affects their crystallinity and material characteristics; PLLA is semicrystalline, while PDLLA is mainly amorphous. 51 Injectable PLLA and PDLLA, although both biostimulatory, differ in collagen formation mechanisms and particle morphology. This leads to varying early-stage volume effects; PLLA demonstrates increasing volume effects over time, while PDLLA produces consistent effects due to different patterns of neotissue growth. 53

PLA's high crystallinity results in reduced flexibility, slow biodegradation, and notable hydrophobicity. 50 An ex vivo study comparing human skin injections of PLLA and PCL microspheres found that PLLA exhibited limited spread after massaging, while PCL showed increased dispersion, highlighting differences in tissue integration. 54 PLA's properties contribute to the formation of implant nodules, a significant concern with intradermal injections, particularly noted with the initial PLLA product, Sculptra (Galderma, Lausanne, Switzerland). An early study reported noninflammatory nodules (2–4 mm) in 12 of 94 cases using intradermal PLLA, appearing 2 to 9 months postinjection. 55 To address nodule formation, a clinical protocol was developed, including higher volume dilution, fewer vials per session, subcutaneous rather than dermal injections, a minimum of 6 weeks between sessions, and postinjection massage. 52 56 A recent U.S. retrospective study across multiple centers, involving 4,483 treatments in 1,002 subjects, found that only 0.4% reported PLLA nodules, 57 indicating that adherence to the subcutaneous injection protocol effectively reduces nodule risk.

The rising demand for intradermal PLA injections, driven by their limitations in enhancing skin texture through deeper injections alone, 15 has led dermatologists, especially those specializing in “skin boosting” with PLA, to explore intradermal injections. Recent studies show that PLA not only promotes collagen production but also induces angiogenesis 58 and offers immune modulation. 59 To reduce the risk of nodule formation from intradermal PLA injections, innovative approaches have been proposed. Lin et al 60 suggest using “super thin” PDLLA suspensions, reconstituted with 12 to 24 mL of sterile water, for shallow wrinkles and skin rejuvenation. Hong et al 61 achieved significant improvements in atrophic acne scars using sonicated PLLA particles of approximately 40 μm, which also prevented nodule formation, potentially attributed to the precise sizing and even distribution of particles achieved through sonication. Korean dermatologists have reported no nodules over 2 years when combining intradermal PLLA with microneedle radiofrequency treatment following topical application. 62 Hyeong et al 63 further confirmed the effectiveness and safety of intradermal poly D lactic acid administration using a microneedle radiofrequency device for treating atrophic acne scars. The majority of these Korean studies have employed Juvelook (VAIM, Seoul, Korea), a PDLLA product mixed with non-cross-linked HA.

It is important to note that even small PLA particles have the potential to obstruct blood vessels, causing tissue ischemia. While rare, it is essential for injectors to recognize this side effect, as arterial blockage by PLA particles can lead to skin necrosis or, in extreme cases, blindness, as reported in cases of cosmetic injection involving PLLA 64 or PDLLA. 65

### Polycaprolactone

PCL is a semicrystalline, aliphatic, water-insoluble polyester, 50 known for its biocompatibility, biodegradability, nontoxicity, and ductility. 66 Its hydrolytically liable ester linkages cause slow hydrolytic degradation. 50 Kim's study 67 indicated that a single intradermal PCL injection increased temporal and facial skin thickness by 27% and 21%, respectively, after 1 year, suggesting long-term ECM remodeling and neocollagenesis. A 4-year study showed that PCL particles maintain 95% of their initial size until the third year, 68 with size reduction and surface texture changes from smooth to rough occurring by the fourth year.

PCL's versatility allows for diverse shapes and sizes, enabling it to mirror ECM properties and support fibroblast growth, cellular migration, adhesion, proliferation, and angiogenesis. 50 The microsphere shape integrates with newly formed collagen type I fibers, forming a sustained network throughout PCL degradation. 69 In animal models, PCL demonstrated a higher increase in fibroblast proliferation compared with calcium hydroxyapatite, and the stimulatory effect on fibroblast proliferation persisted for an extended duration. 70 A study conducted on rat skin showed no significant findings of inflammatory cell infiltration following PCL injections. 71 The microsphere geometry of PCL, particularly the spherical and smooth surface, might have contributed to minimizing inflammatory reactions in tissue responses. 72 Phagocytosis is directly impacted by microsphere size, where smaller particles are swiftly phagocytosed, leading to heightened inflammation. 69 Most injectable PCL products consist of microspheres ranging in size from 25 to 50 μm, offering prolonged protection against phagocytosis. 72 The prolonged biodegradation span of up to 3 years and its water insolubility may be points of concern regarding the long-term safety of PCL, particularly due to its inherent lack of antimicrobial properties. 73

The PCL-based collagen stimulator generally has a favorable safety profile, 74 though there have been reports of late granulomatous reactions. 75 76 A human study on PCL injections showed dermal neocollagenesis accompanied by mild inflammation and foreign body type giant cells, suggesting a necessary level of inflammation for collagen production stimulation. 67 However, excessive inflammatory responses may lead to foreign body granulomas. Nongranulomatous lumps or nodules, often resulting from technical errors like injecting too large boluses or too superficially, are relatively common. Consequently, caution is advised against using PCL-based stimulators in facial areas such as the lips, eyelids, undereye dark circles, and crow's feet lines. 69 For intradermal applications, some practitioners diluted ELLANSE (AQTIS Medical, Utrecht, Netherlands), a known PCL filler, though this off-label use lacks extensive safety validation in the literature.

PCL's hydrophobic nature, leading to inadequate cell adhesion, can be improved by integrating it with polymeric materials like HA and collagen. 66 While PCL is deemed suitable for minor conditions and specific areas, 77 its collagen induction is considered less effective than PLA. 47 Further research is necessary to fully understand PCL-induced neocollagenesis and quantify the collagen production it triggers.

### Polydioxanone

PDO, part of the biodegradable ester-linked polymer family, is characterized by polar, less stable ester bonds that are highly reactive and prone to hydrolysis in tissue. 78 Initially prominent in surgical sutures, PDO's applications have extended to wrinkle reduction using single, coiled, or braided filaments and nonsurgical facelifts employing thick, cogged threads. Recently, PDO has been used into injectable microspheres (ULTRACOL, Ultra V, Seoul, Korea) for volume augmentation and antiwrinkle treatments.

Morphologically, PDO microspheres are distinguished by their irregular surfaces and consistent spherical shapes. This contrasts with PLLA's rough, nonuniform, and pointed structure, and PCL's smooth, uniformly sized spheres. 77 PDO microspheres naturally disperse postinjection, without the need for external manipulation. 79 They exhibit greater biodegradability compared with PLLA and PCL, positioning PDO as potentially the most biodegradable among similar polymers such as PLA and PCL. 77 Postinjection, collagen forms evenly around PDO microparticles without clustering. Over 3 months, the PDO particle area decreases due to degradation, leading to reduced inflammation and cell count, eventually rendering the particles nearly invisible. 79

Most research on PDO currently focuses on threads or mesh forms, leading to a significant gap in detailed laboratory and clinical studies on injectable PDO microspheres. This lack of extensive research challenges the establishment of evidence-based clinical applications for PDO as an injectable skin booster. However, some studies have investigated PDO injections in the skin. A clinical study demonstrated notable improvements in skin gloss, wrinkle reduction, and increased skin density following three PDO microsphere injections. 79 A comparative study by Kwon et al 77 showed that PDO when injected into photoaged mouse skin, induced neocollagenesis and an inflammatory response similar to PLLA and PCL. Another animal study found that injections of both PDO and PLLA resulted in initial increases in collagen types 1 and 3, as well as all three TGF-β subtypes, within 2 weeks. 80 These results indicate that PDO's efficacy in stimulating dermal collagen synthesis may be comparable to that of PLLA or PCL.

## Synthetic Polymers: Mechanisms of Action

The mechanisms of action of biodegradable polymers as skin boosters are primarily focused on their impact on collagen synthesis. PLLA stimulates fibroblast proliferation and reduces collagen-degrading enzymes, thereby increasing collagen and elastin in aged mouse skin. 59 In vivo human skin studies following PDLLA injection showed significant increases in collagen and elastic fibers in the dermis. 81 After injecting PLLA or PDO, there was an initial rise in Col1α1, Col3α1, TGF-β1, TGF-β2, and TGF-β3 isoforms within 2 weeks, followed by a decrease at 12 weeks. PDO showed a more significant increase in Col1α1, Col3α1, TGF-β2, and TGF-β3 than PLLA, whereas PLLA had a higher surge in TGF-β1, indicating its potential advantage in early atrophic scar treatment. 80

**Fig. 4:**
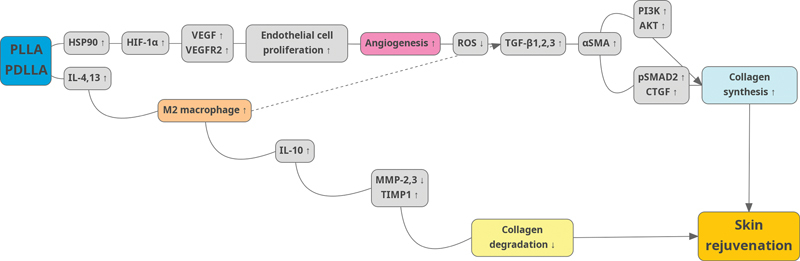
Schematic representation of the proposed mechanisms through which poly-(lactic acid) enhances collagen synthesis in the dermis. PDLLA, poly-(D,L)-lactic acid; PLLA, poly-(L)-lactic acid; TIMP-1, tissue inhibitor of MMP-1.

Macrophage reactions to biostimulatory substances are critical in fibroblast activity and collagen production. In vitro, PLLA triggers an inflammatory response, upregulating inflammation-related cytokines like chemokine ligand 1(CCL1), tumor necrosis factor receptor II (TNFR2), and macrophage inflammatory protein alpha (MIP-1α), and IL-8 in M1 macrophages, while inducing a noninflammatory reaction. In M2 macrophages, PLLA notably upregulates MIP-1α and MIP-1β compared with calcium hydroxylapatite and unstimulated controls. 82 Oh et al 83 found that PDLLA injections enhance collagen synthesis by increasing NRF2 expression in macrophages, which stimulates adipose-derived stem cell proliferation and TGF-β and FGF2 secretion, thus boosting collagen synthesis and potentially mitigating age-related soft tissue volume loss. Another study showed that PLLA injections induce M2 macrophage polarization and upregulate factors like IL-4, IL-13, and TGF-β, leading to increased collagen synthesis in aged skin. 59 Figure 4 illustrates the proposed mechanisms of PLA in collagen synthesis.

## Other Ingredients

### Glycerol

Traditionally, skin-boosting practices have focused on delivering HA into the dermis. Recently, efforts to enhance HA's skin-boosting effects have included incorporating additional ingredients like glycerol, mannitol, and polysaccharides, leveraging their hydrophilic properties. 4 A notable example is BELOTERO Revive (Merz Aesthetics, Frankfurt, Germany), which combines HA and glycerol, showing significant improvements in skin hydration, elasticity, roughness, and tone. 84 A randomized study with 159 participants exhibiting early facial sun damage found that intradermal HA–glycerol injections significantly increased skin hydration for up to 16 weeks in multiple-dose recipients, with mild to moderate injection site reactions as the only transient adverse events. The hydration effects lasted up to 9 months post-last injection, especially in individuals with dry skin. 85 This combination has also been effective in improving skin pigmentation, including hemoglobin and melanin levels. 86 87 The inclusion of glycerol in HA is based on findings about aquaglyceroporin AQP3 in mammalian skin epidermis keratinocytes. Mice lacking AQP3 exhibit dry skin and reduced stratum corneum hydration, 88 primarily due to impaired glycerol transport rather than water movement, a phenomenon also confirmed in human skin. 89 Glycerol is nonimmunogenic and has been safely used in clinical settings for conditions like increased intracranial pressure, establishing its safety profile when injected intravenously. 90

### Amino Acids

The role of specific amino acid (AA) mixtures in stimulating collagen synthesis in human organs has gained attention recently. 91 AAs are fundamental for protein synthesis, with collagen production relying on certain precursor AAs necessary for fibroblast activation. 92 Efficient collagen synthesis requires a continuous supply of these AAs in a specific ratio. 92 A novel treatment approach involves injecting an “amino acid functional cluster” consisting of proline, glycine, lysine, and leucine, combined with low-molecular-weight HA. This method aims to stimulate local collagen synthesis through chemotactic signals. 93 A study evaluating an injectable product containing low molecular weight HA and AAs reported aesthetic improvements in facial skin, including increased fibroblast activity, augmented type III reticular collagen production, increased vascularization, and thickened epidermis. 94 While the scientific data are limited, these findings suggest that AA-based injectables positively affect facial skin photoaging, particularly in ECM remodeling. 95

## Polycomponent Products

Recent advancements have seen the development of products combining HA with beneficial components such as vitamins A, C, and E, antioxidants like ferulic acid and lipoic acids, and AAs. 13 96 These multifaceted formulations aim to amplify the treatment's overall benefits and optimize skin rejuvenation. By integrating various components, polycomponent skin boosters provide a comprehensive solution for diverse skin concerns and promote optimal skin health, 17 enhancing fibroblast functionality, stimulating ECM protein synthesis (especially type 1 collagen and elastin), boosting cellular metabolism, and reducing oxidative damage. 17 96 A prominent example is NCTF135HA (Filorga, Paris, France), which includes non-cross-linked HA, vitamins, AAs, mineral salts, coenzymes, and nucleic acids. Used in France since 1978 and Conformité Européene-marked for the European Union in 2007, this product has pioneered the field. Clinical trials have shown its progressive improvements in wrinkles, fine lines, skin tone, and hydration after consecutive intradermal injections. Objective measurements also indicated reduced pore sizes, enhanced skin color uniformity, improved radiance, and increases in dermal density and thickness. 97

In vitro studies have underscored the role of polycomponent injectables in ECM remodeling. Jäger et al 98 found that NCTF135HA supports cell proliferation and increases mRNA expression of type I collagen, matrix metalloproteinase-1 (MMP-1), and tissue inhibitor of MMP-1 (TIMP-1) in fibroblasts over 11 days in a laboratory culture setting. This suggests a balance between collagen degradation by MMP-1 and its production, facilitated by TIMP-1, enabling sustained dermal collagen production. Another study comparing HA-based skin-boosting solutions, one with idebenone and another with HA, vitamins, AAs, minerals, coenzymes, and antioxidants, in 50 women 99 showed significant improvements in aging skin's clinical appearance. A newer solution including AAs, niacinamide, coenzymes, glutathione, and HA was effective in repairing the epidermal basement membrane, reducing oxidative stress, and managing aging-related factors, thereby enhancing skin elasticity and collagen accumulation for rejuvenation. 100 These “cocktail” skin boosters are thought to create an optimal microenvironment for fibroblast activity. 101

## Botulinum Toxin as a Skin-Boosting Agent

Botulinum neurotoxin (BoNT) injections, traditionally used for hyperkinetic wrinkles, have also been effective in enhancing skin elasticity and hydration and reducing erythema. 46 Intradermal BoNT injections, administered as small approximately 20 U/mL droplets, impact superficial motor neurons, sympathetic nerves in glandular tissues, and the nonneuronal cholinergic system. This broad effect leads to a noticeable enhancement in appearance, 102 expanding BoNT's application in cosmetic dermatology. Its role in suppressing neurogenic inflammation further contributes to improved skin quality. 103 104

The efficacy of BoNT treatments can be augmented when combined with HA-based skin boosters. A study comparing BoNT alone to a combination with fillers for forehead and glabellar lines demonstrated the superiority of the combined approach. It provided longer-lasting results, particularly in reducing dynamic wrinkles and glabellar lines, as preferred in self-evaluations by subjects. 105 A similar enhancement in outcomes was observed when combining BoNT for platysmal bands with intradermal HA injections for skin texture and laxity in the neck, offering a safer and more effective alternative to neck rejuvenation. 106 The concurrent application of BoNT and HA injections presented superior improvements in skin hydration, thickness, and aesthetic outcomes, proposing a safer and more effective option for individuals ineligible for surgical neck lifts when contrasted with the use of BoNT alone. 107 A combined strategy involving BoNT, HA, and energy-based devices has been suggested for addressing horizontal neck wrinkles. 108 Pisal's study 109 underscores the combined treatment's effectiveness, safety, and high patient satisfaction.

Some experts suggest using a custom mix of HA and BoNT in a single syringe for skin boosting. An early trial by Kenner 110 involved concurrently administering an HA and BoNT mixture to the upper face, yielding promising aesthetic results. The specific composition of such mixtures can vary. For instance, Kim 111 recommends a blend of 1 mL of lightly cross-linked HA, 1 mL of 40 units of BoNT, and 1 mL of normal saline. This formulation is applied intradermally across numerous facial sites using an automatic injector. Objective measures showed improvements in skin roughness, reduced TEWL, and increased stratum corneum hydration levels. However, this approach has drawbacks. The mixing process may lead to uneven dosing in certain areas and the potential spread of neuromodulators to adjacent muscles, raising concerns about unintended diffusion of the mixture into neighboring tissues. 112

## Delivery Methods

### Intradermal Injection Technique

The intradermal multi-injection method, involving multiple punctures for precise solution delivery, 17 has gained prominence in aesthetic dermatology, especially for cutaneous antiaging treatments. 99 This technique involves microinjections of substances directly into the superficial skin layers, preferably the papillary dermis. 96 It allows active ingredients to interact directly with dermal fibroblasts and keratinocytes, crucial for enhancing the youthful appearance of the skin and influencing metabolic processes. 101 Despite some technical feasibility concerns, intradermal injections are achievable using appropriate products and precise techniques.

**Fig. 5:**
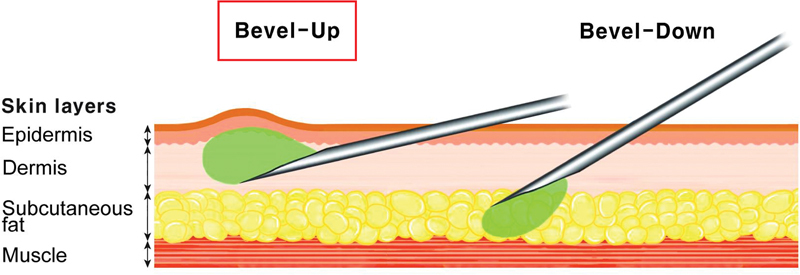
The recommended technique for secure intradermal product placement involves delicately inserting a needle at approximately a 10-degree angle, utilizing a tangential approach to the skin. Employing a 33- or 34-gauge fine needle with its bevel toward the skin's surface ensures the necessary shallow depth for the procedure.

For accurate intradermal placement, inserting the needle at approximately a 10-degree angle in a tangential approach to the skin is recommended. 14 A 33- or 34-gauge fine needle, with its bevel facing the skin's surface, is preferred for achieving the necessary shallow depth ( Fig. 5 ). Practitioners should use a closely spaced multipuncture technique for precision rather than the conventional retrograde method used for deep dermal HA filler injections. 14 Understanding the rheological properties of the skin booster product is key to ensuring correct injection placement and optimal results. 13 There is typically an inverse relationship between the particle size of injected ingredients and their lateral distribution and penetration depth, with smaller particles reaching deeper into the dermis and subcutaneous fat layers. 113

**Fig. 6:**
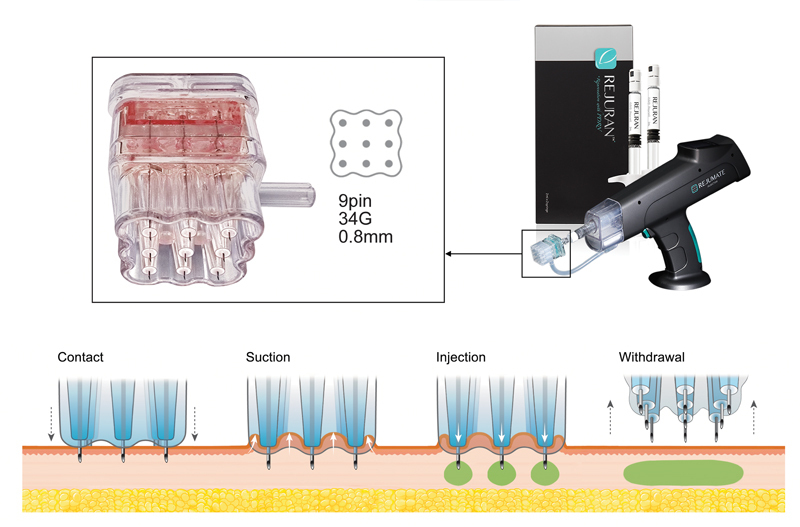
The automatic multineedle injector (REJUMATE, PharmaResearch, Gyeonggi-do, Korea) employs negative pressure suction technology within the microneedle cartridge, ensuring secure needle placement and minimizing product loss during injection.

While conventional intradermal injections using a hypodermic needle are simple and cost-effective, they have drawbacks such as discomfort, needle phobia, potential inconsistencies, and longer treatment durations. To address these issues, alternative methods like multineedle injectors have been developed, enhancing the accuracy and stability of intradermal injections. 114 Innovations like the REJUMATE (PharmaResearch, Gyeonggi-do, Korea), an automatic multineedle injector, employ negative pressure suction technology for secure needle placement and reduced product loss during injection ( Fig. 6 ).

### Needle-free Jet Injectors

To alleviate the pain and discomfort associated with needle penetration, particularly for those with needle phobia, “no-needle injection” devices using compression springs 115 or compressed gas 116 for propulsion have been developed. However, traditional needle-free jet injectors face challenges such as slower injection speeds, imprecise depths and volumes, discomfort from tissue disruption, and longer recovery times. 117 Recently, laser-powered needle-free injectors have emerged as a solution. These devices utilize laser pulses to create vapor bubbles, generating pressure for precise, and tiny-volume injections at specific dermal depths. 118 An example of this technology, MiraJet (JSK Biomed, Seoul, Korea), demonstrates accurate filler distribution, increased clinical effectiveness, reduced discomfort, and fewer side effects, showing great potential for skin rejuvenation treatments. 117

**Fig. 7:**
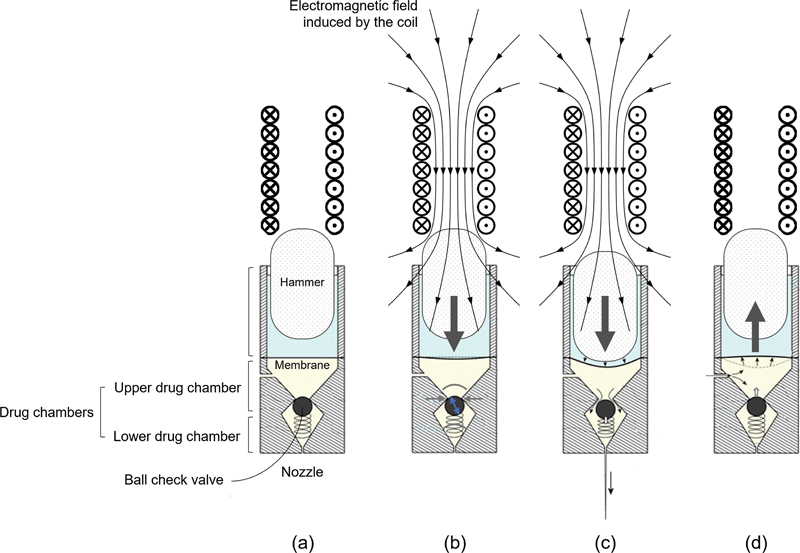
Illustrations demonstrating the operational mechanism of the Curejet (Baz Biomedic, Seoul, Korea), a novel needle-free jet injector utilizing an electromechanical actuator to regulate the piston's movement, facilitating electronic control over liquid displacement and subsequent jet velocity.

While laser-assisted needle-free methods offer advantages, they also have limitations, particularly concerning their penetration depth. 119 An alternative, electromechanical actuators have been introduced to regulate the piston's movement, allowing for electronic control over liquid displacement and jet velocity. 120 An example of this technology is the Curejet (Baz Biomedic, Seoul, Korea), which operates based on the Lorentz force principle ( Fig. 7 ). These electromagnetic force injectors achieve deeper penetration, often reaching several millimeters, making them suitable for administering thicker fluids or gels. 119 This feature makes them an effective option for treating scars or thicker skin tissues, such as the scalp.

## Side Effects

Skin booster injections are generally safe but should be approached with an awareness of potential adverse effects. 17 Common transient reactions include mild erythema and swelling, lasting a few hours postprocedure. Patients may experience pain, discomfort, occasional bruising, or needle marks. Postinflammatory hyperpigmentation is rare. Vascular compromise is a significant concern, and practitioners should identify high-risk areas before injection. Rarely, PLA can lead to serious vascular accidents, including visual loss. 121

Superficial injections of polymers might cause small to medium-sized papules or nodules. The size and duration of these lumps vary by product. Non-cross-linked HA and PN typically result in small, transient lumps, whereas synthetic polymers like PLA can cause more noticeable, longer-lasting lumps. Immediate massage post-PLA injection can help resolve implant nodules. 17 If untreated, nodules may become harder to dissolve over time. While nonsurgical treatments are available, their effectiveness varies. 122 Corticosteroid injections can cause the “donut effect,” leading to tissue atrophy and increased nodule visibility, hence are best avoided. Injecting HA fillers around the nodule may reduce its appearance. Noninflammatory nodules that are palpable but not visible may naturally resolve within 2 years, so immediate treatment is not always necessary. 123 High-frequency ultrasound can be used as a noninvasive method to monitor PLLA degradation and the development of papules and nodules. 124

Although rare, complications, such as foreign body granuloma formation, characterized by inflammatory nodules, should be acknowledged as potential risks of skin booster injections. 17 To minimize inflammation, it is recommended to schedule energy-based device treatments either a few weeks after booster injections or conduct these procedures beforehand. 13 A significant concern arises when substances approved only for topical are directly injected, as this can introduce immunogenic particles into the dermis. This practice may lead to local or systemic hypersensitivity reactions, including foreign-body granulomas. 125 Given the growing popularity of skin booster injections in cosmetic procedures, clinicians must remain vigilant about these potential adverse effects. It is crucial for practitioners to restrict the use of skin boosters to products that are specifically approved for injectable use in humans.

## Limitations

Despite growing interest in skin booster injections among physicians and patients, several limitations exist. Standardization of skin booster materials and procedures is needed for consistent outcomes across different demographics. The difficulty in objective measurements complicates result comparison, and the necessity for multiple sessions may deter cost-sensitive patients. Furthermore, the limited number of evidence-based controlled studies challenges the predictability of outcomes, especially with combination “cocktails.” 13 Understanding the interaction and stability of mixed ingredients is crucial, yet lacks substantial evidence-based support.

## Conclusion

Injectable skin boosters focus on enhancing aesthetics by improving skin quality, seeking to restore a healthy, radiant, and hydrated complexion rather than just mechanical effects. Biopolymers, synthetic polymers, AAs, and polycomponent products find widespread use in cosmetic medicine and surgery. Combining skin boosters with other treatments enhances outcomes, but requires careful consideration for safe and effective skin restoration. The scarcity of specific scientific data limits progress in this field, affecting understanding and development. Future research should focus on larger controlled studies with objective assessments and histopathology to establish optimal protocols, booster combinations, delivery techniques, and new treatment indications. Further basic research is needed to elucidate mechanisms, effects on skin components, immune modulation, impacts on cellular aging, and clinical efficacy.
